# Loss of exosomal miR‐26a‐5p contributes to endometrial cancer lymphangiogenesis and lymphatic metastasis

**DOI:** 10.1002/ctm2.846

**Published:** 2022-05-11

**Authors:** Jing Wang, Xiaodi Gong, Linlin Yang, Lijuan Li, Xiaoyan Gao, Ting Ni, Xiaoming Yang, Qiong Fan, Xiao Sun, Yudong Wang

**Affiliations:** ^1^ Department of Gynecologic Oncology The International Peace Maternity and Child Health Hospital School of Medicine Shanghai Jiao Tong University Shanghai China; ^2^ Shanghai Municipal Key Clinical Specialty Shanghai China; ^3^ Shanghai Key Laboratory of Embryo Original Disease Shanghai China

Dear Editor,

1

Lymphatic metastasis is one of the important parameters used for predicting prognosis for endometrial cancer (EC). The increased peri‐tumoural lymphatic vessels density is correlated with metastasis and a poor outcome.^1^, ^2^, ^3^ It is necessary to explore the potential target and identify the specific mechanism that promotes this process in EC. In this study, the results provide the translational pathway via which exosomal miR‐26a‐5p contributes to lymph node metastasis (LNM), and could serve as a specific target of the treatment in EC.

Scientific evidence shows that exosomal miRNAs are potential biomarkers of cancer patients.^4^, ^5^ To explore potential exosomal miRNAs in plasma of EC patients, we utilised next‐generation sequencing (NGS) (Figure S1). Table S1 showed the top 10 significantly dysregulated plasma exosomal miRNAs from patients with EC. Plasma exosomal miR‐26a‐5p (exo‐miR‐26a‐5p) level was obviously decreased in patients suffering EC, especially in those with LNM in comparison with that in the controls (Figure 1A). Exo‐miR‐26a‐5p from patients with LNM showed significantly reduced, compared to those without LNM, whereas other nine miRNAs didn't (Figure S2). As shown in Table S2, plasma exo‐miR‐26a‐5p level was correlated to LNM and FIGO stage in EC patients. Compared to normal endometrial tissue, miR‐26a‐5p level was substantially decreased in EC lesions. Moreover, it had a positive relation with plasma exo‐miR‐26a‐5p (Figure 1B, C). Analysis of the cancer genome atlas (TCGA) data indicated a consistent result (Figure S3A–C). The increased density of peri‐tumoural lymphatic endothelial hyaluronan receptor‐1 (LYVE‐1) in patients with LNM was negatively related to miR‐26a‐5p level in the original lesions, indicating that miR‐26a‐5p downexpression may induce LNM of EC (Figure 1D, E).

**FIGURE 1:**
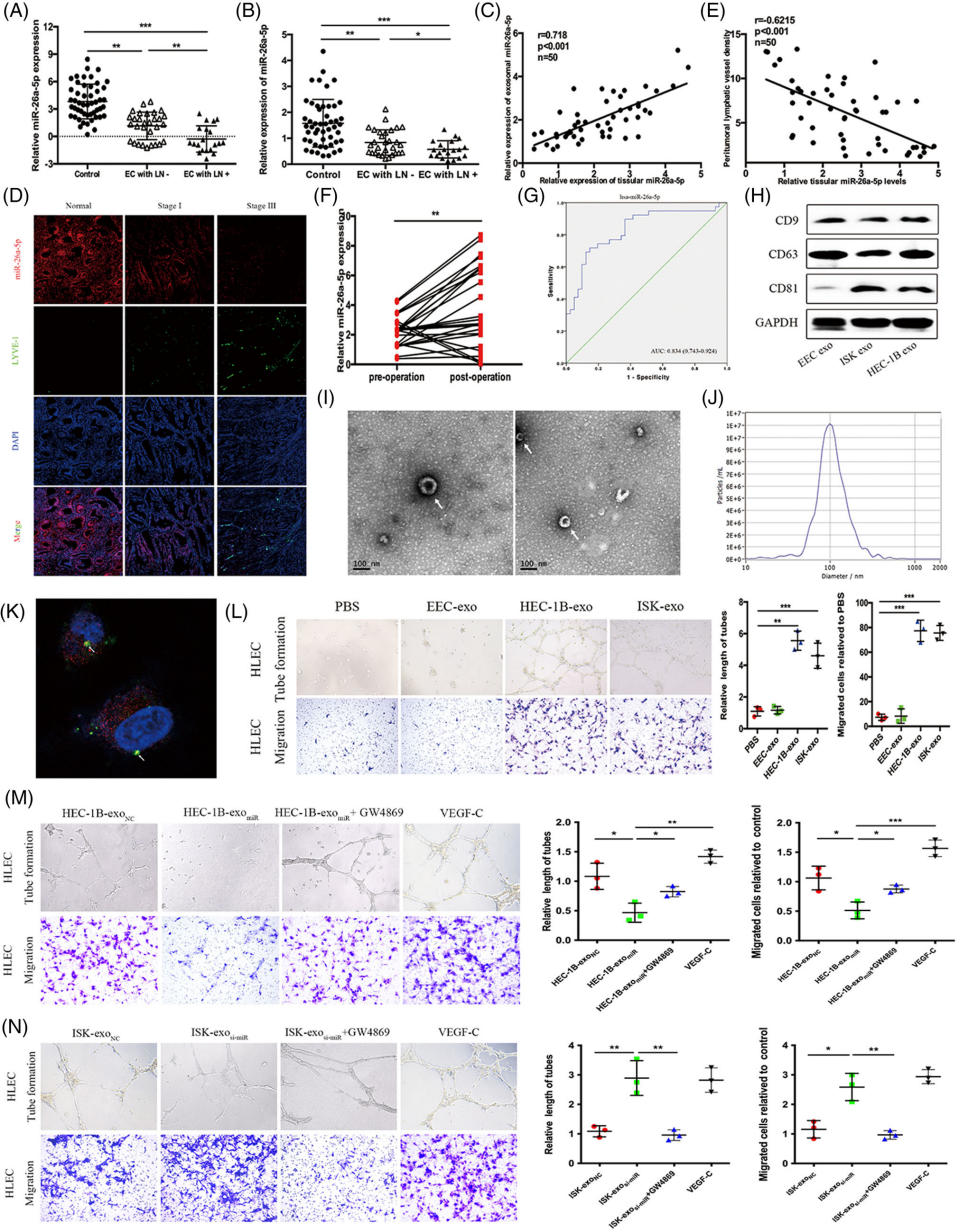
Plasma exosomal miR‐26a‐5p is associated with LNM in EC patients, and miR‐26a‐5p transferred to HLECs promotes lymphangiogenesis in vitro. (A) qRT‐PCR analysis to detect plasma exosomal miR‐26a‐5p levels in healthy controls and EC patients. EC with LN‐: endometrial cancer with lymph node negative; EC with LN+: endometrial cancer with lymph node positive. (B) qRT‐PCR analysis of miR‐26a‐5p expression in normal endometrial tissue and EC tissue. (C) Pearson correlation analysis between miR‐26a‐5p levels of EC tissues and miR‐26a‐5p levels of plasma exosomes from EC patients. (D) FISH images of miR‐26a‐5p and LYVE‐1 in paraffin‐embedded normal endometrial tissue and EC tissue with or without LNM. (E) Pearson correlation between miR‐26a‐5p expression and LYVE‐1 expression in EC tissue. (F) Comparison of plasma exosomal miR‐26a‐5p levels (*n* = 25) between pre‐operative and post‐operative EC patients. (G) ROC curve analyses to evaluate the potential of plasma exosomal miR‐26a‐5p for diagnosis of LNM in EC patients. (H‐J) Purified exosomes were identified via western blot, transmission electron microscopy, and NanoSight analysis. (K) Representative images of Cy3 and PKH67 fluorescence in HLECs after a 48 h exposure to HEC‐1B cell‐derived PKH67‐labelled exosomes. (L) Representative images of tube formation and cell migration of HLECs after treatment with PBS, and exo. (M) Micrographs of tube formation and cell migration in HLECs treated with HEC‐1B‐exo_NC_, HEC‐1B‐exo_miR_, HEC‐1B‐exo_miR_+GW4869 or VEGF‐C, VEGF‐C as a positive control for HEC‐1B‐exo_miR_ treatment. (N) Micrographs of tube formation and cell migration in HLECs treated with ISK‐exo_NC_, ISK‐exo_si‐miR,_ ISK‐exo_si‐miR_+GW4869 or VEGF‐C, VEGF‐C as a positive control for ISK‐exo_si‐miR_ treatment. Mean ± SD are provided. *p < .05, **p < .01, ***p < .001

Compared to that in patients before operation, significantly higher level of plasma exo‐miR‐26a‐5p was found in patients after the operation, indicating a correlation with EC lesions (Figure 1F). Exo‐miR‐26a‐5p had a relatively high diagnostic value with an area under the curve of .834 in discriminating EC patients with LNM (Figure 1G). We extracted exosomes from the medium of EC cells and confirmed their identity (Figure 1H–J). We found decreased miR‐26a‐5p levels in EC cells. Moreover, it had a lower abundance in HEC‐1B‐exo than that in non‐carcinoma endometrial epithelial cells (EEC)‐exo. Compared to control, exo‐miR‐26a‐5p levels from EEC transfected with miRNA inhibitor were remarkably reduced, and compared to incubation with EEC‐exo, miR‐26a‐5p levels in human lymphatic endothelial cells (HLECs) were also reduced by EC cell‐exo treatment (Figure S4A–D). Cy3‐labelled miR‐26a‐5p mimics transfected HEC‐1B, and then HLECs were incubated with PKH67‐labelled HEC‐1B‐exo. Fluorescence collocated in HLECs indicated that HLECs internalised HEC‐1B‐exo (Figure 1K, Figure S4E). We found that EC cells‐exo treatment enhanced HLECs lymphangiogenesis and migration ability (Figure 1L). HEC‐1B‐exo_miR_ (miR‐26a‐5p‐overexpressing) failed to induce migration and tube formation by HLECs, whereas pre‐treatment with GW4869, an inhibitor of exosome secretion, significantly reversed these changes. Similarly, ISK‐exo_si‐miR_ (miR‐26a‐5p‐silenced) strongly enhanced HLECs lymphangiogenesis and migration ability, whereas GW4869 pre‐treatment abolished these effects. Compared to VEGF‐C treatment, as positive control, HEC‐1B‐exo_miR_ significantly reduced the migration and tube formation abilities of HLECs, whereas ISK‐exo_si‐miR_ didn't (Figure 1M, N).

As Figure S5 shown, miR‐26a‐5p inhibited EC cells proliferation, migration and invasion. A subcutaneous tumour model^6^ demonstrated that HEC‐1B‐exo_miR_ reduced tumour growth, and the tumours had smaller size and weight, and Ki67 expression was lower than the controls (Figure 2A–E). A popliteal lymphatic model illustrated that HEC‐1B‐exo_miR_ remarkably reduced HEC‐1B cell metastasis to the lymph node, and that the volume and weight of footpad tumours were significantly lower than those of the controls (Figure 2F–J). Luciferase IHC staining showed decreased positive lymph node in the HEC‐1B‐exo_miR_ group, indicating that increase in exo‐miR‐26a‐5p remarkably attenuated the cell migration capacity (Figure 2K). Treatment with HEC‐1B‐exo_miR_ significantly enhanced miR‐26a‐5p level in peri‐tumoural lymphatics compared to treatment with HEC‐1B‐exo_vector_ or PBS (Figure 2L).

**FIGURE 2:**
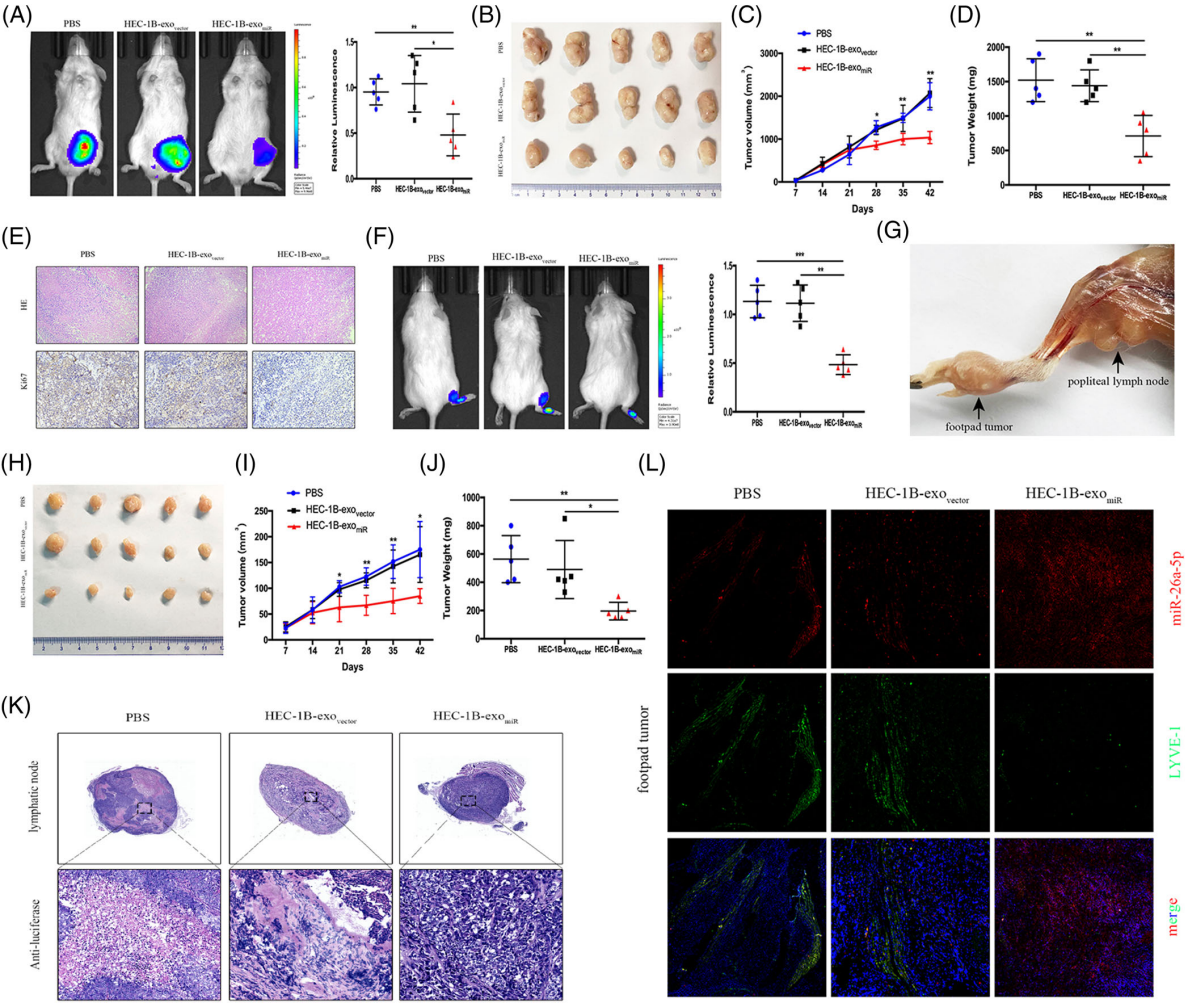
Exosomal miR‐26a‐5p inhibits EC tumour proliferation and LNM in vivo. (A) Representative bioluminescence images and histogram analysis of subcutaneous tumours in NOD‐SCID mice, which treated with PBS, HEC‐1B‐exo_vector_, or HEC‐1B‐exo_miR_ (*n* = 5). (B) Images of subcutaneous tumours in mice treated with PBS, HEC‐1B‐exo_vector_, or HEC‐1B‐exo_miR_ (*n* = 5). (C, D) The tumour volumes and weights (*n* = 5). (E) Representative HE and immunohistochemical staining images demonstrating Ki67 expression. (F) Bioluminescence images and analysis of popliteal metastatic lymph node in NOD‐SCID mice, which treated with PBS, HEC‐1B‐exo_vector_, or HEC‐1B‐exo_miR_ after HEC‐1B cell inoculation into the footpad (*n* = 5). (G) Representative image of the popliteal lymph node. (H) Representative images of footpad tumours. (I, J) The measured footpad tumour volumes and weights (*n* = 5). (K) IHC of anti‐luciferase for popliteal lymph node treated with PBS or the indicated exosomes. Luciferase‐positive tumour cell in lymph node indicated metastasis. (L) Staining of miR‐26a‐5p and LYVE‐1 mice footpad tumour sections. Mean ± SD are provided. *p < .05, **p < .01, ***p < .001

To illustrate how miR‐26a‐5p regulates lymphangiogenesis, miRNA target prediction algorithms were employed to determine the target gene. LEF1 was determined as a putative target associated with lymphatic metastasis in EC. The luciferase activity of 3′‐untranslated regions of LEF1 could be weaken by miR‐26a‐5p (Figure 3A, B). Ectopic miR‐26a‐5p downregulated the protein and mRNA levels of LEF1, which were reverted by silencing miR‐26a‐5p (Figure 3C). Ectopic miR‐26a‐5p remarkably reduced c‐myc, β‐catenin and VEGFA levels, whereas LEF1 restoration abolished the effects. GW4869 pre‐treatment rescued β‐catenin, LEF1, c‐myc and VEGFA expression (Figure 3D). The biological effects of exo‐miR‐26a‐5p could be reversed by LEF1 upregulation, as evaluated by cell migration and tube formation experiments (Figure 3E).

**FIGURE 3:**
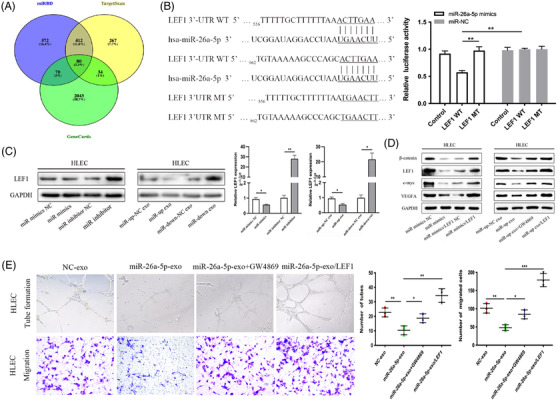
EC‐secreted exosomal miR‐26a‐5p targets LEF1 induced lymphatic vessel formation in HLECs. (A) miR‐26a‐5p target predictions identified from different databases (miRDB, TargetScan and GeneCards). (B) Sequence alignment between miR‐26a‐5p and the 3′‐UTR of LEF1 (left), and effects of miR‐26a‐5p mimics/NC on the luciferase reporter activity in wild type (WT) and mutant type (MT) (right). (C) Protein and mRNA levels of LEF1 assessed through western blotting and qRT‐PCR, respectively, in HLECs, after transfection with miR‐26a‐5p mimics/inhibitor or indicated exosomes. (D) β‐catenin, LEF1, c‐myc and VEGFA protein levels were detected by western blotting in HLECs treated with an miR‐26a‐5p mimics, indicated exosomes from GW4869‐pre‐treated EC cells, or in the presence of LEF1 overexpression plasmid. (E) Upregulation of LEF1 reversed the biological effects of exosomal miR‐26a‐5p, as evaluated by tube formation and cell migration experiments. Mean ± SD are provided (*n* = 3). *p < .05, **p < .01, ***p < .001

Growing evidence supports transcription factors (TFs) play vital roles in tumour metastasis.^7^ In this study, RNA sequencing was performed for EEC and EC cells. Intersection analysis indicated that three TFs might directly control miR‐26a‐5p expression (Figure 4A, B). Specifically, we found transcription factor EB (TFEB) levels were significantly reduced in EC cells compared with EEC cells. A similar result was found for pre‐miR‐26a‐5p‐1 (pre‐1) and pre‐miR‐26a‐5p‐2 (pre‐2) level (Figure 4C–E). TFEB overexpression enhanced miR‐26a‐5p, pre‐1 and pre‐2 level in EC cells, which was reversed by downregulation of TFEB. HEC‐1B transfected by plasmid encoding TFEB gene substantially affected exo‐miR‐26a‐5p level (Figure 4F, G). The results revealed that HLECs incubated with HEC‐1B_TFEBov_‐exo reduced migration capacity of HLECs, while HEC‐1B_TFEBsi_‐exo enhanced (Figure 4H). To validate the binding site of TFEB to the promoters of pre‐1 and pre‐2, chromatin immunoprecipitation (ChIP)‐PCR assay was employed, which indicated strong enrichment of TFEB (Figure 4I). Following the ChIP assay, the Southern blot of pre‐1 and pre‐2 exhibited segments that were detected by anti‐TFEB antibodies (Figure 4J). The luciferase reporter assay demonstrated that TFEB could substantially enhance the activities of pre‐1 and pre‐2 promoter reporters (Figure 4K). Collectively, these findings provide support that TFEB regulates miR‐26a‐5p expression and that exo‐miR‐26a‐5p derived from EC cells could be absorbed by HLECs and may promote lymphatic vessel formation via LEF1/c‐myc/VEGFA axis (Figure 4L).

**FIGURE 4:**
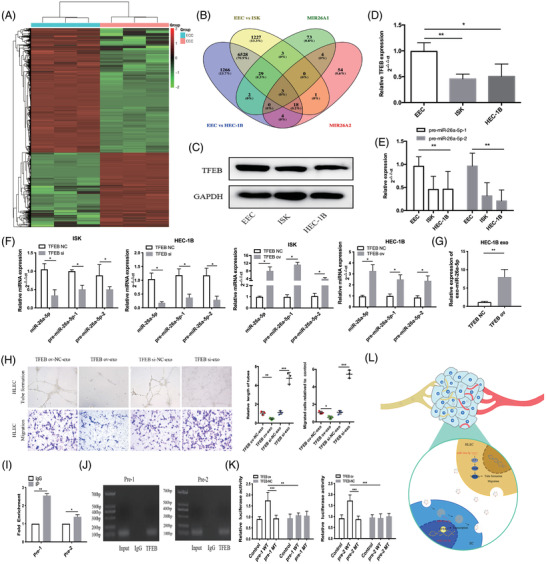
Transcription factor EB controls miR‐26a‐5p expression levels. (A) Heatmap of differentially expressed mRNAs between EEC and EC cells. ECC: endometrial cancer cell; EEC: endometrial epithelial cells. (B) The intersection between differential mRNAs and putative direct transcription factors of miR‐26a‐5p, as predicted by the JASPAR database. MIR26A1: the predicted transcription factors of pre‐1; MIR26A2: the predicted transcription factors of pre‐2. (C, D) Protein and mRNA levels of TFEB in EC and EEC cells respectively. (E) pre‐1 and pre‐2 levels in EC cells and EEC cells. (F) Levels of miR‐26a‐5p, pre‐1, and pre‐2 after TFEB overexpression/downexpression plasmids were transfected into EC cells. (G) mRNA levels of miR‐26a‐5p in HEC‐1B‐derived exosomes after transfection with TFEB overexpression plasmid. (H) Representative pictures of cell migration and tube formation in HLECs treated with HEC‐1B‐derived exosomes after transfection with TFEB overexpression/downexpression plasmids, compared with that in HLECs treated with NC‐exo. (I) The relative expression of segments containing TFEB binding sites detected via qRT‐PCR following ChIP assay. (J) Southern blot of indicated segments with anti‐TFEB antibody following ChIP assay. (K) Relative luciferase activity of pre‐1 and pre‐2 in HEC‐1B after transfection with luciferase reporter including wild type (WT) or mutant type (MT). (L) Illustrative model of EC cell‐secreted exosomal miR‐26a‐5p absorbed by HLECs and associated promotion of lymphangiogenesis via activation of the LEF1/c‐myc/VEGFA axis. Mean ± SD are provided (*n* = 3). *p < .05, **p < .01, ***p < .001

In conclusion, the results provide a new understanding that low plasma exo‐miR‐26a‐5p levels are related to LNM in patients suffering EC. EC cells‐secreted miR‐26a‐5p‐devoid exosomes absorbed by HLECs could induce lymphatic vessel formation via the activation of LEF1 and could be helpful for early identification of EC patients with LNM.

## CONFLICT OF INTEREST

The authors declare that no conflict of interests.

## Supporting information

Supporting information.
**FIGURE S1**. Heatmap shows the dysregulated expression of miRNAs. N: healthy donors; EC: endometrial cancer patients

Supporting information.
**FIGURE S2**. Top 10 dysregulated plasma exosomal miRNAs in EC patients (50 healthy controls, 30 patients without LNM, 20 patients with LNM). *p < .05, **p < .01, ***p < .001

Supporting information.
**FIGURE S3**. Analysis of miR‐26a‐5p expression in EC tissues from TCGA data (A, B) Analysis of miR‐26a‐5p expression in EC tumour tissues and paracancerous tissues from TCGA data, respectively. (C) ROC curve analysis to evaluate the diagnostic potential of miR‐26a‐5p as a marker for EC from TCGA data.

Supporting information.
**FIGURE S4**. EC‐secreted miR‐26a‐5p absorbed by human lymphatic endothelial cells. (A) qRT‐PCR analysis of miR‐26a‐5p expression in EEC and EC cells. (B) Comparisons of miR‐26a‐5p levels, detected via qRT‐PCR, in HEC‐1B cells and paired exosomes with EEC levels. (C) exo‐miR‐26a‐5p levels from EEC transfected with vector, inhibitor NC or miR‐26a‐5p inhibitor. (D) miR‐26a‐5p expression levels in HLECs treated with exosomes from EEC, HEC‐1B and ISK for 24 h. (E) Representative images of Cy3 and PKH67 fluorescence in HLECs after 48 h of incubation. Mean ± SD are provided (*n* = 3). *p < .05, **p < .01, ***p < .001

Supporting information.
**FIGURE S5**. miR‐26a‐5p inhibited EC cell growth, invasion and migration. (A) Cell viability was detected using CCK‐8 assay. (B) Cell proliferation was measured using colony formation assay. (C) Cell migration ability was detected by wound healing assay. (D) Cell migration and invasive abilities were measured using transwell assay. *p < .05, **p < .01, ***p < .001

Supporting information.
**TABLE S1**. The top 10 significantly dysregulated miRNAs in EC plasma exosomes compared with those in healthy donors

Supporting information.
**TABLE S2**. Correlation between clinical parameters and the expression levels of plasma exosomal miR‐26a‐5p in patients with EC
