# Supplementary figures and images for: Loss of exosomal miR‐26a‐5p contributes to endometrial cancer lymphangiogenesis and lymphatic metastasis
Source: Clin Transl Med. 2022 May 11;12(5):e846. doi: 10.1002/ctm2.846 (PMC9092006; doi:10.1002/ctm2.846)

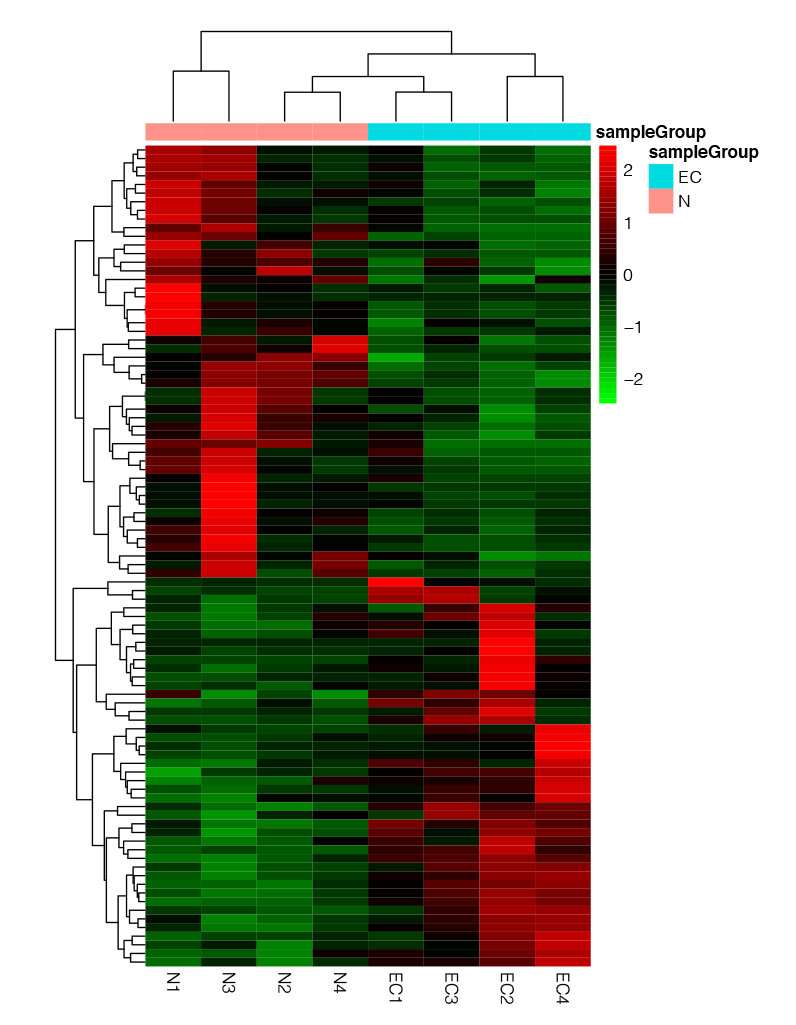

Supplement: Supplementary file 1 — Supporting information. FIGURE S1. Heatmap shows the dysregulated expression of miRNAs. N: healthy donors; EC: endometrial cancer patients [file CTM2-12-e846-s005.tif]

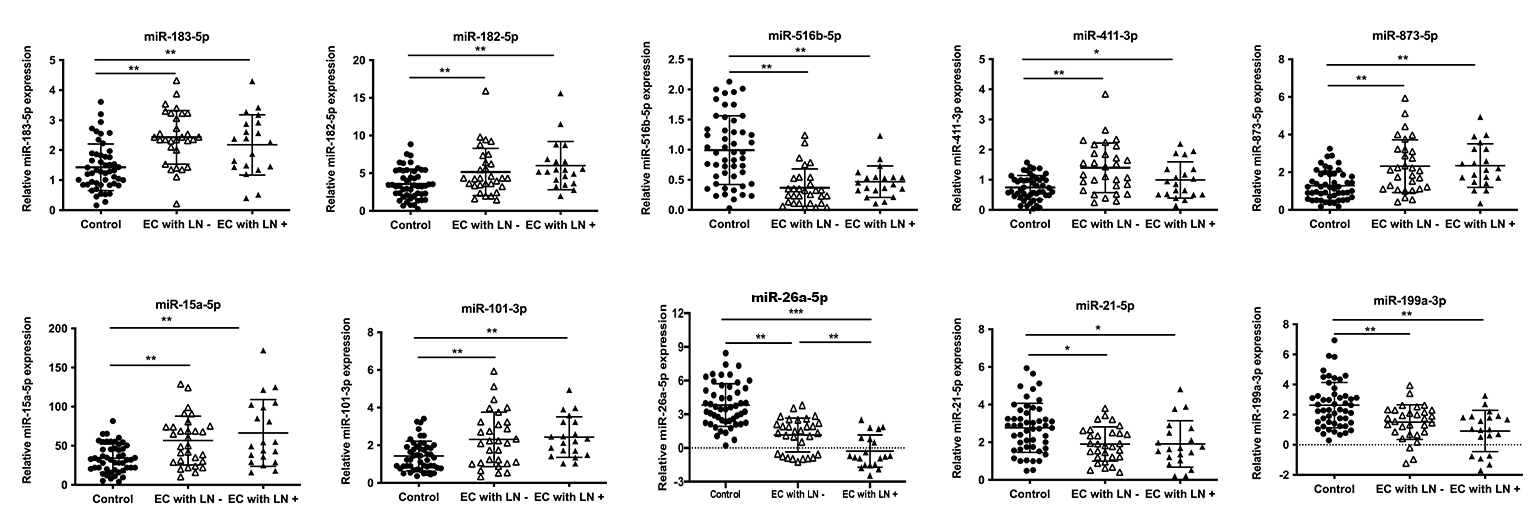

Supplement: Supplementary file 2 — Supporting information. FIGURE S2. Top 10 dysregulated plasma exosomal miRNAs in EC patients (50 healthy controls, 30 patients without LNM, 20 patients with LNM). *p < .05, **p < .01, ***p < .001 [file CTM2-12-e846-s002.tif]

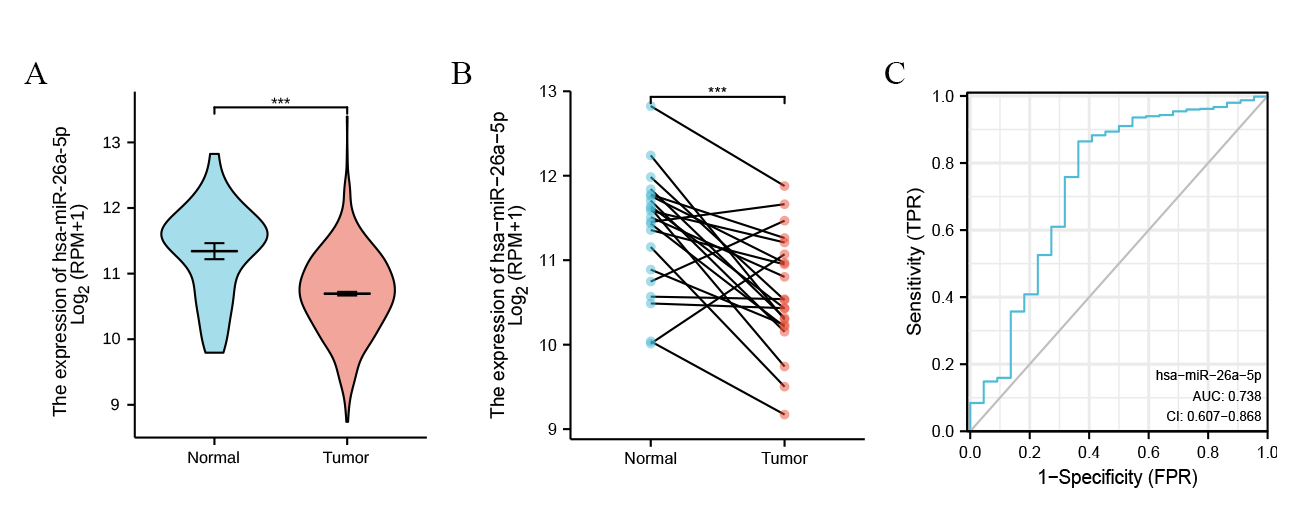

Supplement: Supplementary file 3 — Supporting information. FIGURE S3. Analysis of miR‐26a‐5p expression in EC tissues from TCGA data (A, B) Analysis of miR‐26a‐5p expression in EC tumour tissues and paracancerous tissues from TCGA data, respectively. (C) ROC curve analysis to evaluate the diagnostic potential of miR‐26a‐5p as a marker for EC from TCGA data. [file CTM2-12-e846-s006.tiff]

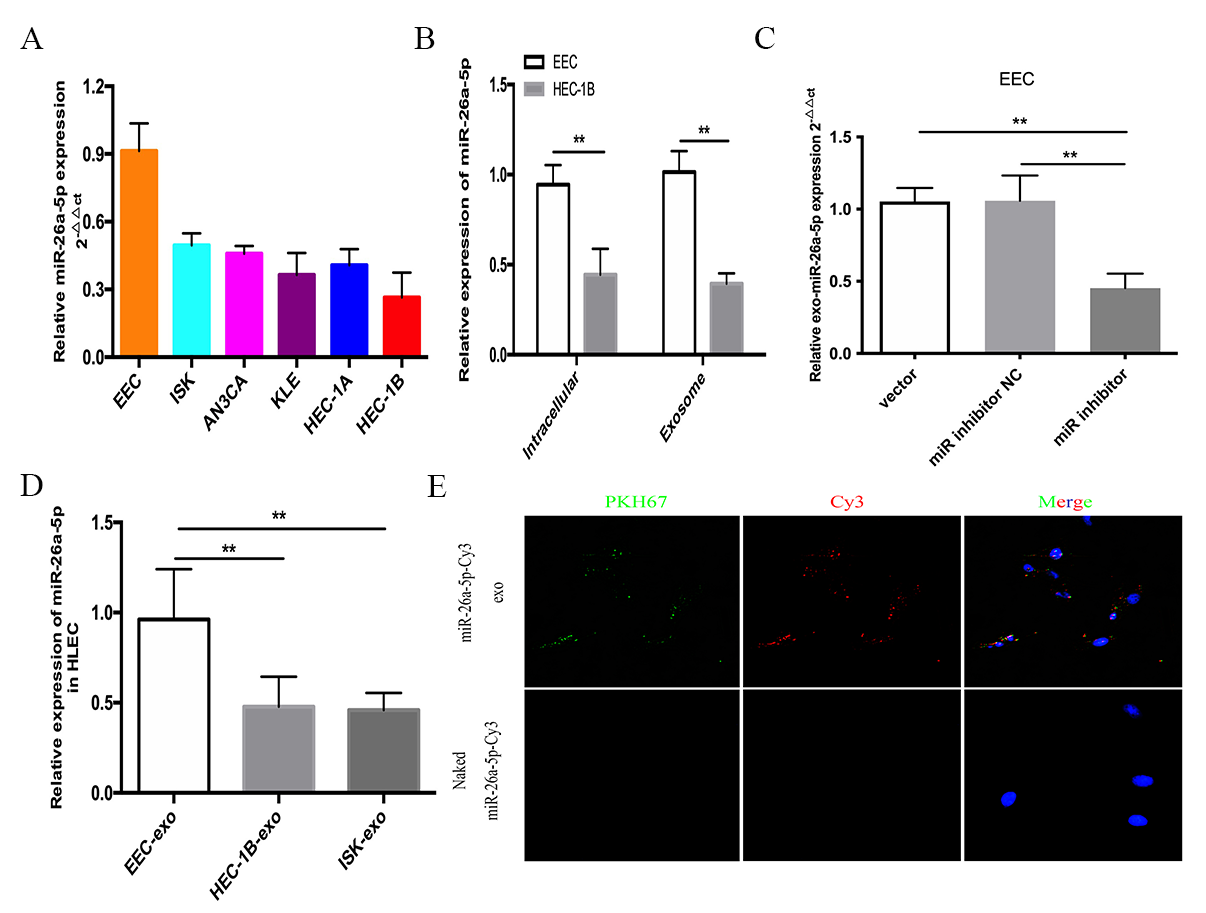

Supplement: Supplementary file 4 — Supporting information. FIGURE S4. EC‐secreted miR‐26a‐5p absorbed by human lymphatic endothelial cells. (A) qRT‐PCR analysis of miR‐26a‐5p expression in EEC and EC cells. (B) Comparisons of miR‐26a‐5p levels, detected via qRT‐PCR, in HEC‐1B cells and paired exosomes with EEC levels. (C) exo‐miR‐26a‐5p levels from EEC transfected with vector, inhibitor NC or miR‐26a‐5p inhibitor. (D) miR‐26a‐5p expression levels in HLECs treated with exosomes from EEC, HEC‐1B and ISK for 24 h. (E) Representative images of Cy3 and PKH67 fluorescence in HLECs after 48 h of incubation. Mean ± SD are provided (n = 3). *p < .05, **p < .01, ***p < .001 [file CTM2-12-e846-s004.tif]

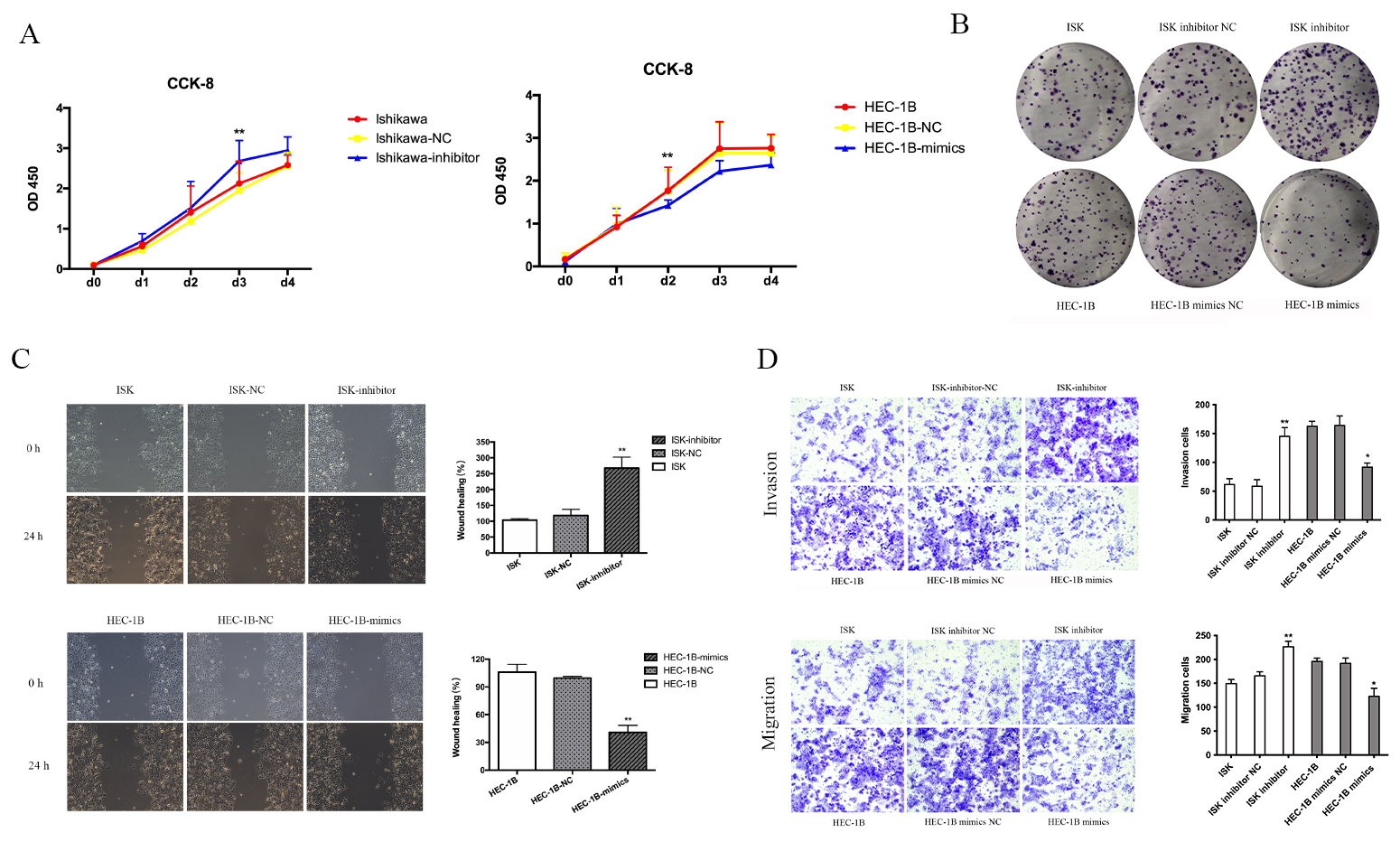

Supplement: Supplementary file 5 — Supporting information. FIGURE S5. miR‐26a‐5p inhibited EC cell growth, invasion and migration. (A) Cell viability was detected using CCK‐8 assay. (B) Cell proliferation was measured using colony formation assay. (C) Cell migration ability was detected by wound healing assay. (D) Cell migration and invasive abilities were measured using transwell assay. *p < .05, **p < .01, ***p < .001 [file CTM2-12-e846-s001.tif]

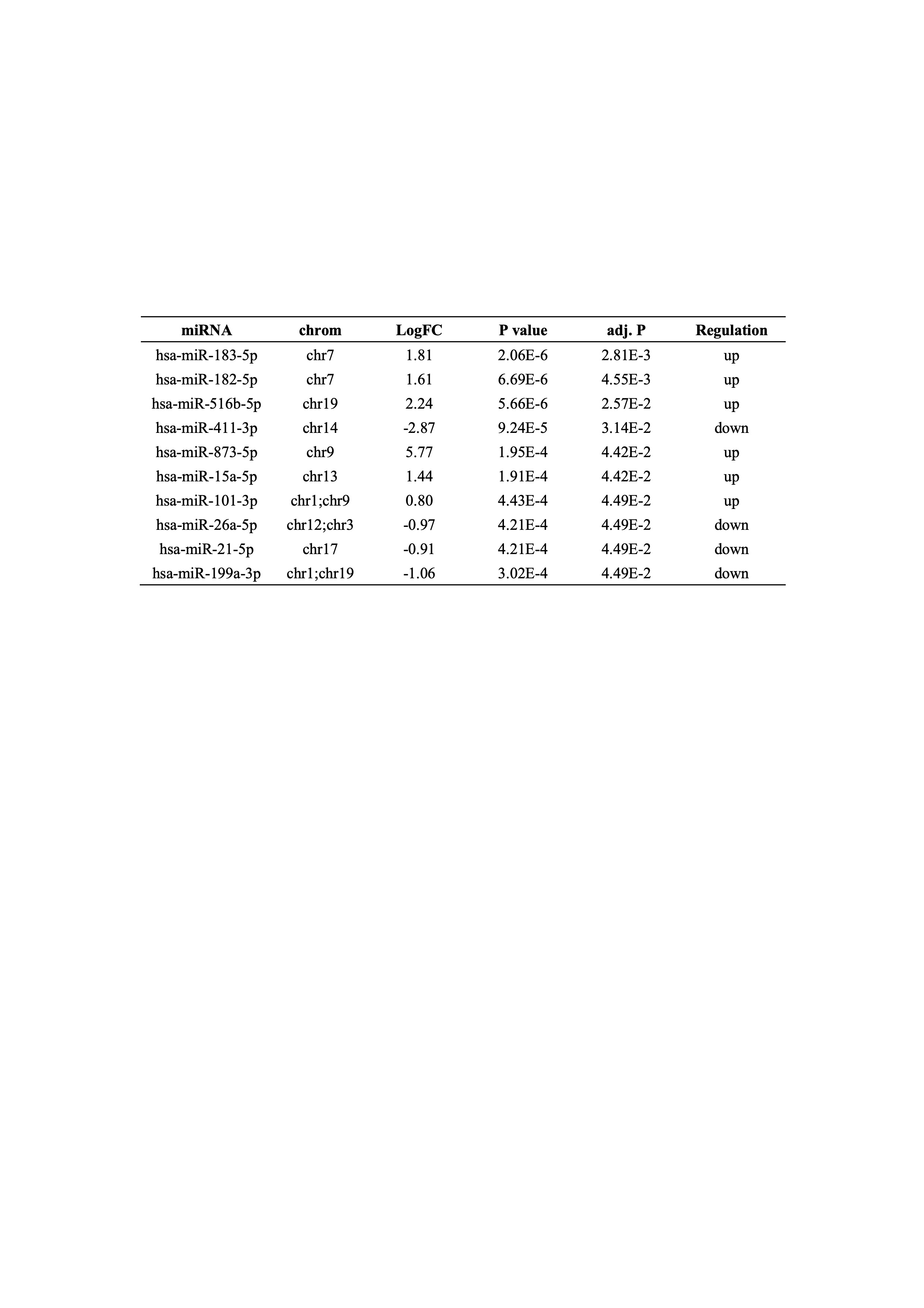

Supplement: Supplementary file 6 — Supporting information. TABLE S1. The top 10 significantly dysregulated miRNAs in EC plasma exosomes compared with those in healthy donors [file CTM2-12-e846-s003.jpg]

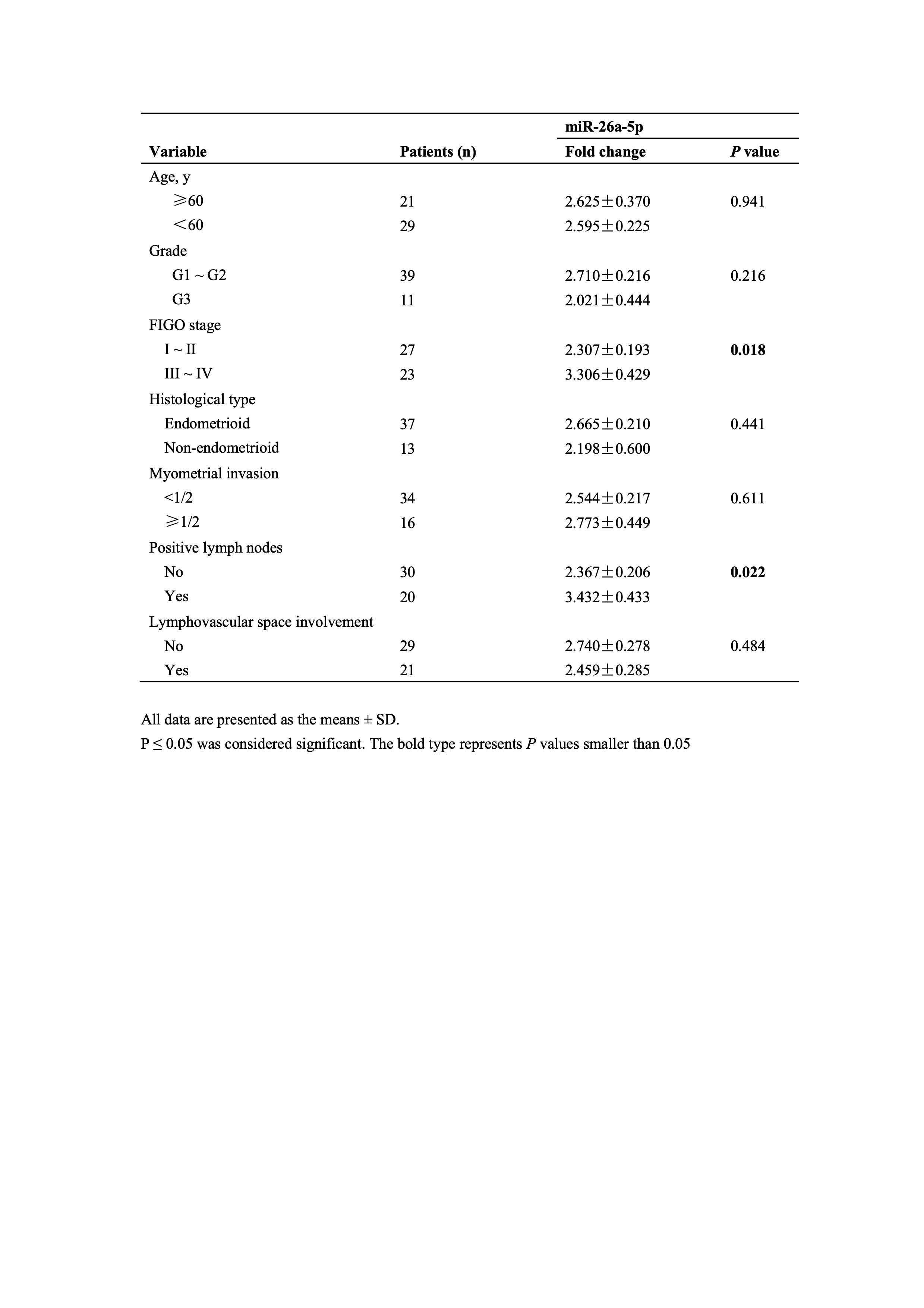

Supplement: Supplementary file 7 — Supporting information. TABLE S2. Correlation between clinical parameters and the expression levels of plasma exosomal miR‐26a‐5p in patients with EC [file CTM2-12-e846-s007.jpg]
